# Prevalence and anatomical location of muscle tenderness in adults with nonspecific neck/shoulder pain

**DOI:** 10.1186/1471-2474-12-169

**Published:** 2011-07-22

**Authors:** Lars L Andersen, Klaus Hansen, Ole S Mortensen, Mette K Zebis

**Affiliations:** 1 National Research Centre for the Working Environment, Lersø Parkalle 105, DK 2100 Copenhagen Ø, Denmark; 2 Department of Occupational and Environmental Medicine, Bispebjerg University Hospital, Bispebjerg Bakke DK 2400 Copenhagen, Denmark; 3 Institute of Sport Sciences and Clinical Biomechanics, University of Southern Denmark, DK 5320 Odense M, Denmark

## Abstract

**Background:**

Many adults experience bothersome neck/shoulder pain. While research and treatment strategies often focus on the upper trapezius, other neck/shoulder muscles may be affected as well. The aim of the present study is to evaluate the prevalence and anatomical location of muscle tenderness in adults with nonspecific neck/shoulder pain.

**Methods:**

Clinical neck/shoulder examination at two large office workplaces in Copenhagen, Denmark. 174 women and 24 men (aged 25-65 years) with nonspecific neck/shoulder pain for a duration of at least 30 days during the previous year and a pain intensity of at least 2 on a modified VAS-scale of 0-10 participated. Exclusion criteria were traumatic injuries or other serious chronic disease. Using a standardized finger pressure of 2 kg, palpable tenderness were performed of eight anatomical neck/shoulder locations in the left and right side on a scale of 'no tenderness', 'some tenderness' and 'severe tenderness'.

**Results:**

In women, the levator scapulae, neck extensors and infraspinatus showed the highest prevalence of severe tenderness (18-30%). In comparison, the prevalence of severe tenderness in the upper trapezius, occipital border and supraspinatus was 13-19%. Severe tenderness of the medial deltoid was least prevalent (0-1%). In men, the prevalence of severe tenderness in the levator scapulae was 13-21%, and ranged between 0-8% in the remainder of the examined anatomical locations.

**Conclusions:**

A high prevalence of tenderness exists in several anatomical locations of the neck/shoulder complex among adults with nonspecific neck/shoulder pain. Future research should focus on several neck/shoulder muscles, including the levator scapulae, neck extensors and infraspinatus, and not only the upper trapezius.

**Trial Registration:**

ISRCTN60264809

## Background

A high prevalence of upper extremity pain exists among adults working in sedentary occupations [1]. Neck/shoulder pain is a risk factor for long-term sickness absence among white-collar workers [2], and every other office worker experience neck/shoulder pain on a weekly basis [1,3]. Pain symptoms are believed to worsen in response to prolonged static muscle activity and/or repetitive job tasks [4,5], causing muscle metabolic disturbances [6].

Kaergaard and coworkers found a strong correlation between reported neck/shoulder pain and clinically verified muscle tenderness [7]. Especially, tenderness of the upper trapezius muscle often co-exists with neck/shoulder pain [6,8,9]. The upper trapezius muscle is - due to its bulky and superficial nature - well suited for clinical research, and displays clear physiological differences between symptomatic and non-symptomatic individuals regarding electromyographic activity [10,11], muscle strength [10,11], muscle fiber morphology [12], stem cell content [13], and intramuscular metabolites [14]. In spite of the inordinate focus on the upper trapezius, other muscles of the neck/shoulder complex may be affected as well - and should therefore not be overlooked when treating neck/shoulder pain.

Ohlsson and coworkers developed a clinical protocol for diagnosing disorders of the neck, shoulder and arm - e.g. tension neck syndrome, frozen shoulder and lateral epicondylitis [15]. Juul-Kristensen and coworkers extended this protocol, and included the diagnosis of trapezius myalgia - frequent neck pain with co-existing tenderness and tightness of the upper trapezius muscle [8]. The overall prevalence of these disorders is low to modest [8,15]. Together these studies show that many people experience non-specific neck/shoulder pain, i.e. pain in absence of the aforementioned clinically diagnosed disorders.

The aim of the present study is to evaluate the prevalence and anatomical location of muscle tenderness among adults with nonspecific neck/shoulder pain, but without traumatic injuries or other serious chronic disease.

## Methods

### Participants

**Figure 1:**
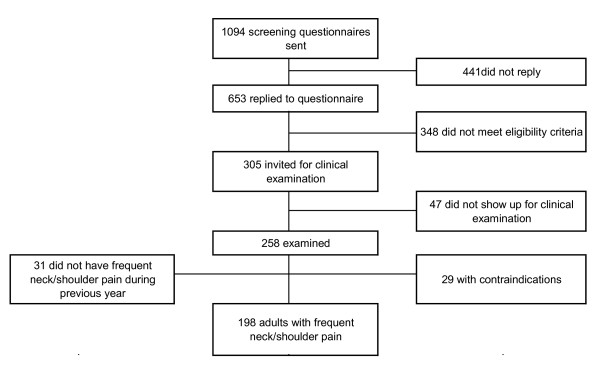
**Flow of participants**.

We obtained data for this prevalence-study as part of a randomized controlled trial [16], register number ISRCTN60264809. A screening questionnaire to locate generally healthy adults with frequent nonspecific neck/shoulder pain went out to 1094 employees from two large office companies, and 653 replied (60%). Figure 1 outlines the flow of participants. Exclusion criteria for participation in the investigation were a medical history of cardiovascular or cerebrovascular accident or disease (n = 8), fibromyalgia (n = 1), rheumatoid arthritis (n = 7), cervical disc herniation (n = 5), whiplash (n = 18), pregnancy (n = 16), working less than 30 hours per week (n = 69), performing more than 2 hours per week of vigorous physical exercise (n = 95), or declining to participate in the study (n = 206). Based on the screening questionnaire replies employees with an average neck/shoulder pain intensity during the last 3 months of at least 2 on a modified VAS scale of 0-10 [17,18], neck/shoulder tenderness of at least 'some' (scale of 'no', 'some' and 'severe' tenderness), and a duration of at least 30 days with neck/shoulder pain during the last year [19] were invited for a clinical neck/shoulder examination (n = 305) (47% of those who replied to the questionnaire). Out of the 305 invited 258 presented for the examination.

To locate details that were not evident from the screening questionnaire the clinical examination started with an interview on medical history and pain symptoms, measurement of blood pressure, Hawkins test for subacromial impingement [20], and Spurling's foramen compression test for cervical radiculopathy [21]. Exclusion criteria during the clinical examination were hypertension above 160/100, a positive Hawkins test, a positive Spurling's test or a medical history of traumatic neck/shoulder injuries or other serious chronic disease. This procedure lead to exclusion of 60 participants; hypertension above 160/100 (n = 6), severe neck/shoulder trauma (n = 9), subacromial impingement syndrome (n = 2), cervical radiculopathy (n = 2), myasthenia (n = 1), severe pain due to osteoarthritis (n = 2), disc herniation (n = 1), withdrawal of consent (n = 5). Further, we asked the participants if they had experienced neck/shoulder pain frequently during the previous year. This lead to exclusion of another 31 participants, who had experienced only a brief period of pain during the previous year, for example, due to hectic sporting activities. The remaining 198 generally healthy adults with frequent nonspecific neck/shoulder pain formed the basis for this study.

### Examination for tenderness

A team of four trained examiners (physical therapists) performed the examination for tenderness. The examiners were blinded to the questionnaire replies on self-reported pain symptoms. The examination built on a previously described procedure from our laboratory by Juul-Kristensen and coworkers, who reported good reliability of palpable tenderness scores of the neck/shoulder muscles [8]. Due to the subjective nature of manual palpation tests, we used several strategies to improve the internal validity of our study. For manual palpation tests to be valid a standardized finger pressure needs to be applied. Using a scale and a pinch grip dynamometer, the examiners practiced a finger-pressure (thumb, index finger, and middle finger, respectively) and pinch grip (i.e. pressing the thumb against the index finger) of 2 kg - a procedure which was repeated frequently between examinations during the study period. Repeating this procedure frequently all four examiners were able to apply a finger pressure and pinch grip ranging from 1.8 to 2.2 kg without looking at the scale or dynamometer. Twenty volunteers from our department participated during a pilot week for the examiners to become mutually calibrated with the manual palpation procedure. At the end of the pilot week the inter-rater reliability for the tenderness scores was high between all four examiners (ICC > 0.70). Further, as previously reported, test-retest reliability of the tenderness score in 64 of the present participants, who was re-tested on a later occasion, was good (Intraclass Correlation Coefficient (ICC) = 0.88) [22].

**Figure 2:**
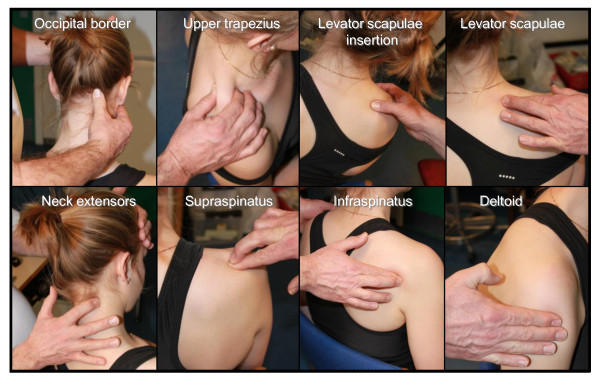
**Palpation of the eight examined neck/shoulder locations (only illustrated for the right side)**.

The examiners determined tenderness by deep palpation of eight anatomical neck/shoulder locations on the left and right side. Based on the participants response and feedback during the palpation, the examiner used a score of 0-2, corresponding to 'no tenderness', 'some tenderness' or 'severe tenderness', respectively, for each anatomical location (Figure 2):

#### Occipital border

Using the thumb, the examiners palpated the entire occipital border along the linea nuchalis superior - the tenderest part along the border defined the score. The examiner supported the forehead of the participant with the contralateral hand.

#### Upper trapezius

Using a pinch grip with the thumb and index finger, the examiner palpated the upper trapezius from the acromion to the border of neck - not including the vertical part of the neck. The tenderest part along the trapezius determined the tenderness score.

#### Levator scapulae insertion

Using the thumb, the examiner palpated the insertion-spot of the levator scapulae at the superior angle of scapulae.

#### Levator scapulae

Using the index finger with reinforcement from the middle finger, the examiner palpated the levator scapulae from the insertion of the scapulae until the sixth cervical vertebrae. The tenderest part along the levator scapulae defined the tenderness score.

#### Neck extensors

Using the index- and middle finger, the examiner palpated the neck extensor muscles all the way from the sixth cervical vertebrae to below the occipital border. The examiner supported the forehead of the participant with the contralateral hand. The tenderest part along the extensor muscles defined the tenderness score.

#### Supraspinatus

Using the index finger with reinforcement from the middle finger, the examiner palpated the hollow where the acromion and clavicula joins.

#### Infraspinatus

Using the index finger with reinforcement from the middle finger, the examiner palpated the spot where the infraspinatus is superficial - below the posterior deltoid lateral to the medial trapezius. To accurately locate the infraspinatus the examiner first asked the participant to actively perform an external rotation of the arm and then to relax.

#### Medial deltoid

Using the index and middle fingers, the examiner palpated the medial deltoid from below the acromion to the insertion at humerus. The tenderest part along the muscle determined the tenderness score.

### Statistics

We used the SAS statistical software (SAS institute, Cary, NC, version 9.2) for all analyses. Using the GENMOD procedure, one-way analysis of variance (ANOVA) determined differences in tenderness scores between the examined anatomical locations (averaged for left and right side). We also performed the ANOVA to determine differences in tenderness scores between genders.

Further, we calculated the weighted kappa coefficient between the tenderness scores to test for possible clustering of tenderness between anatomical locations.

Finally, we performed a multiple regression analysis with backward elimination to determine which anatomical location was most strongly correlated to perceived pain intensity. Tenderness scores of the 8 anatomical locations - averaged for left and right side - were entered in the model as explanatory variables. The outcome was questionnaire-based neck/shoulder pain intensity during the last week (scale 0-10). The analysis was controlled for gender.

Results are reported as frequencies or means (SD) for descriptive data, and least square means (SE) from the ANOVA output. We accepted P-values of 0.05 or less as statistically significant.

## Results

Table 1 shows demographics, clinical and work-related characteristics of the participants. The duration and intensity of reported neck/shoulder pain was high, and participants spent most of their working time at a computer. The women had higher tenderness scores in the neck/shoulder muscles and lower blood pressure than the men.

**Table 1:** Demographics, clinical and work-related characteristics

	Women (n = 174)	Men (n = 24)	P-value
Demographics			
Age, year	43 (11)	45 (10)	0.31
Height, cm	168 (6)	182 (8)	<0.0001
Weight, kg	67 (12)	83 (13)	<0.0001
Body Mass Index, kg^.^m^-2^	24 (4)	25 (4)	0.14
Clinical			
Days with neck/shoulder pain previous year	191 (116)	147 (111)	0.08
Neck/shoulder pain intensity previous 3 months, scale 0-10	5.1 (2.1)	4.5 (1.6)	0.21
Total Tenderness Score, scale 0-32	13 (5)	9 (4)	<0.0001
Systolic blood pressure mmHg	124 (13)	137 (13)	<0.0001
Diastolic blood pressure mmHg	83 (9)	89 (9)	0.003
Work-related			
Computer use, percentage of worktime	94 (13)	91 (18)	0.42
Weekly working hours	38 (4)	38 (6)	0.79
Duration of office work, years	11 (10)	12 (11)	0.64

Demographics, clinical and work-related characteristics of the 198 generally healthy office workers with frequent neck/shoulder pain. Total Tenderness Score is the sum of tenderness scores of 'no' (= 0), 'some' (= 1), and 'severe' (= 2) tenderness of the 8 investigated neck/shoulder anatomical locations of the left and right side (scale 0-32).

Table 2 shows the prevalence of tenderness and average tenderness scores with significance levels from the ANOVA between anatomical locations and genders. For women, the prevalence of tenderness (some + severe) was above 50% in all of the examined anatomical locations but the deltoid muscle. The prevalence of severe tenderness ranged between 18-30% in the infraspinatus, neck extensors and levator scapulae, and ranged between 13-19% in the upper trapezius, occipital border, and supraspinatus. Only 1 out of 174 woman experienced severe tenderness in the medial deltoid. Because the prevalence of severe tenderness was comparable in the levator scapula muscle and levator scapulae insertion (Table 2) we simply use the term "levator scapulae" in the following.

**Table 2:** Prevalence of tenderness

		Women (n = 174)					Men (n = 24)					
					
		Prevalence of tenderness (%)			Tenderness score (0-2)	Between-site difference	Prevalence of tenderness (%)			Tenderness score (0-2)	Between-site difference	Between-gender difference (P-value)
Site		Some	Severe	Total	LSmeans (SE)		Some	Severe	Total	LSmeans (SE)		
Neck extensors	R	48.9	27.6	76.4	1.01 (0.05)	* # $ £	50.0	4.2	54.2	0.56 (0.10)	*	<0.0001
	L	47.1	25.3	72.4			45.8	4.2	50.0			

Levator scapulae insertion	R	52.3	26.4	78.7	0.99 (0.04)	* # $ £	75.0	16.7	91.7	1.00 (0.08)	* # $ £ &	0.93
	L	57.5	17.8	75.3			66.7	12.5	79.2			

Levator scapulae	R	49.4	23.6	73.0	0.99 (0.05)	* # $ £	45.8	12.5	58.3	0.83 (0.12)	* # &	0.23
	L	56.9	22.4	79.3			54.2	20.8	75.0			

Infraspinatus	R	32.8	30.5	63.2	0.89 (0.05)	* #	25.0	4.2	29.2	0.31 (0.09)	* $ £	<0.0001
	L	43.7	20.1	63.8			29.2	0.0	29.2			

Upper trapezius	R	60.3	12.6	73.0	0.89 (0.04)	* #	50.0	8.3	58.3	0.67 (0.10)	* &	0.03
	L	60.9	16.1	77.0			58.3	4.2	62.5			

Occipital border	R	55.2	13.8	69.0	0.84 (0.04)	*	45.8	4.2	50.0	0.65 (0.10)	* &	0.07
	L	56.3	14.9	71.3			58.3	8.3	66.7			

Supraspinatus	R	39.7	19.0	58.6	0.74 (0.05)	*	41.7	4.2	45.8	0.50 (0.10)	*	0.03
	L	44.3	12.6	56.9			50.0	0.0	50.0			

Medial deltoid	R	12.1	0.6	12.6	0.10 (0.02)		0.0	0.0	0.0	0.04 (0.03)	*	0.08
	L	5.8	0.0	5.8			8.3	0.0	8.3			

Prevalence of 'some tenderness' and 'severe tenderness' - as well as the total prevalence of tenderness (total = some + severe; note that not all sums match due to rounding) - of 8 selected anatomical locations in the 198 generally healthy office workers with frequent neck/shoulder pain. The anatomical locations are ranked in descending order of the tenderness score for women. Average scores (0-2) are calculated as the average right and left score, where some tenderness = 1 and severe tenderness = 2. Between-location and between-gender differences were based on ANOVA's of the tenderness scores (0-2).

*: sign.diff. from deltoideus

#: sign.diff. from supraspinatus

$: sign.diff. from occipital border

£: sign.diff. from trapezius

&: sign.diff. from infraspinatus.

The ANOVA also showed that average tenderness scores in men were significantly lower than in women in the neck extensors (P < 0.0001), infraspinatus (P < 0.0001), upper trapezius (P = 0.03), and supraspinatus (P = 0.03). In men, the prevalence of severe tenderness in the levator scapulae was 13-21%, and ranged between 0-8% in the remainder of the examined anatomical locations.

On an exploratory basis, we also performed the ANOVA for tenderness with *side *(left and right) as a covariate. However, there was no main effect of *side*, i.e. tenderness scores were not significantly different between the left and right sides.

Table 3 shows that the association of tenderness between the examined anatomical locations was generally weak. The left and right side of each examined anatomical location generally showed the highest level of agreement (0.33 - 0.54).

**Table 3:** Weighted kappa coefficients

		Occipital border		Upper trapezius		Levator scapulae insertion		Levator scapulae		Neck extensors		Supra- spinatus		Infra- spinatus		Medial deltoid
		**R**	**L**	**R**	**L**	**R**	**L**	**R**	**L**	**R**	**L**	**R**	**L**	**R**	**L**	**R**
Occipital border	R		0.47	0.22	0.19	0.18	0.12	0.23	0.17	0.32	0.27	0.07	0.05	0.13	0.12	0.03
	L	0.47		0.11	0.07	0.21	0.16	0.30	0.21	0.28	0.29	0.09	0.11	0.12	0.17	0.03
Upper trapezius	R	0.22	0.11		0.41	0.07	0.14	0.29	0.21	0.17	0.13	0.08	0.03	0.09	0.08	0.07
	L	0.19	0.07	0.41		0.05	0.16	0.17	0.25	0.10	0.19	0.07	0.13	0.18	0.11	0.02
Levator scapulae insertion	R	0.18	0.21	0.07	0.05		0.33	0.39	0.21	0.18	0.19	0.17	0.16	0.17	0.16	0.03
	L	0.12	0.16	0.14	0.16	0.33		0.28	0.40	0.11	0.11	0.14	0.16	0.25	0.13	0.01
Levator scapulae	R	0.23	0.30	0.29	0.17	0.39	0.28		0.46	0.24	0.21	0.19	0.15	0.15	0.18	0.07
	L	0.17	0.21	0.21	0.25	0.21	0.40	0.46		0.20	0.24	0.17	0.14	0.21	0.15	0.01
Neck extensors	R	0.32	0.28	0.17	0.10	0.18	0.11	0.24	0.20		0.54	0.10	0.07	0.20	0.12	0.05
	L	0.27	0.29	0.13	0.19	0.19	0.11	0.21	0.24	0.54		0.10	0.15	0.18	0.15	0.04
Supraspinatus	R	0.07	0.09	0.08	0.07	0.17	0.14	0.19	0.17	0.10	0.10		0.45	0.22	0.20	0.06
	L	0.05	0.11	0.03	0.13	0.16	0.16	0.15	0.14	0.07	0.15	0.45		0.20	0.22	0.00
Infraspinatus	R	0.13	0.12	0.09	0.18	0.17	0.25	0.15	0.21	0.20	0.18	0.22	0.20		0.52	0.01
	L	0.12	0.17	0.08	0.11	0.16	0.13	0.18	0.15	0.12	0.15	0.20	0.22	0.52		0.03
Medial deltoid	R	0.03	0.03	0.07	0.02	0.03	0.01	0.07	0.01	0.05	0.04	0.06	0.00	0.01	0.03	

Weighted kappa coefficients between the investigated neck/shoulder locations, pooled for men and women (n = 198).

The multiple regression analysis with backward elimination showed that tenderness in the levator scapulae (β = 0.22, P < 0.01), infraspinatus (β = 0.16, P < 0.05) and deltoid (β = 0.17, P < 0.05) remained significant in the final model for neck/shoulder pain intensity.

## Discussion

Our study shows a high prevalence of tenderness in several anatomical locations of the neck/shoulder complex among generally healthy adults with nonspecific neck/shoulder pain. Tenderness scores were highest in the levator scapulae and neck extensors in women, and highest in the levator scapulae in men.

The upper trapezius is a large superficial muscle extending from the occipital bone and cervical vertebraes to the acromion and lateral part of the clavicle [23]. Much research on neck/shoulder pain has focused on the upper trapezius muscle [6,8,9]. Although trapezius myalgia - chronic tenderness and tightness of the upper trapezius muscle - is the most common clinical diagnosis in adults with self-reported neck/shoulder pain [8], our results show that severe tenderness more commonly occurs in the levator scapulae, neck extensors and infraspinatus. Although the levator scapulae, neck extensors and infraspinatus are smaller than the trapezius, future research should focus also on these muscles and not only the upper trapezius.

The levator scapulae origins from the upper cervical vertebraes, extend along the back of the neck and inserts at the medial angle of scapulae [23]. The neck extensor muscles - e.g. semispinalis and splenius - extend along the back of the neck [23]. Together the levator scapulae and neck extensor muscles provide stability and prevent forward flexion and rotation of the neck during static work positions, e.g. at the computer. Although our study did not investigate the underlying mechanisms of tenderness, prolonged muscle fiber activation of the levator scapulae and neck extensors during long hours of computer work may lead to development of pain and tenderness [6].

Our study shows that a high prevalence of infraspinatus tenderness also exists in adults with nonspecific neck/shoulder pain. The infraspinatus externally rotates the shoulder and provides stability and motion of the arm during many work tasks [24]. During most types of computer work, e.g. when using the mouse or typing at the keyboard, the humerus is slightly externally rotated, which may put excessive stress on the infraspinatus [25] and potentially lead to development of tenderness.

Although more sophisticated methods for research studies exists, e.g. digitalized pressure algometry [26], physical therapists primarily rely on their hands for manually diagnosing upper extremity disorders. As many of the investigated neck/shoulder muscles overlap anatomically - e.g. the trapezius muscles covers both the suprasinatus and levator scapulae - completely differentiating tenderness between muscles may not be possible. Nevertheless, the low kappa coefficients between the investigated anatomical neck/shoulder locations suggest that using the present method of manual palpation only minor overlap of tenderness exists. The weighted kappa coefficients of table 3 show no systematic clustering of tenderness among the different anatomical locations of the neck/shoulder complex. As the method of manual palpation using a pre-learned pressure provides quick and reliable information on muscle tenderness [8,16], therapists may use the present screening tool to determine specific muscle tenderness in patients with neck/shoulder pain and thereby more efficiently target rehabilitation exercises.

Our study is the first to report on the prevalence and anatomical location of palpable muscle tenderness among men with neck/shoulder pain. In spite of the limited male sample size, we showed clear gender differences for the prevalence of examiner-verified tenderness in spite of comparable subjective pain symptoms. While many women suffered from severe tenderness in several of the investigated muscles, the levator scapulae was the primary source of severe tenderness in men. Women generally have lower pressure pain thresholds than men [27,28], likely due to more potent neural inhibitory control mechanisms in men [29]. Thus, using a standardized finger pressure of 2 kg as in our study may lead to stronger sensitivity of pain in women than men in spite of comparable questionnaire replies on neck/shoulder pain intensity. This suggests that application of these results to the general population should take gender into account.

The multiple regression analysis showed that tenderness of the levator scapula, infraspinatus and deltoid were significantly related to perceived neck/shoulder pain intensity. While this finding was not surprising regarding the infraspinatus and levator scapulae (i.e. both showed a high prevalence of tenderness), the significant influence of deltoid tenderness on perceived pain intensity was unexpected. Speculatively, tenderness of the deltoid muscle may reflect referred pain from undiagnosed progressing disease, e.g. shoulder joint osteoarthritis. Altogether, tenderness of the levator scapulae, infraspinatus and deltoid appears to be interesting areas for future neck/shoulder research.

### Limitations

A limitation is the small number of men (n = 24), which increases the risk of statistical type II errors. Also, the anatomical overlap of several neck/shoulder muscles - e.g. trapezius and supraspinatus - may weaken the ability to precisely determine tenderness of specific muscles. Further, the study would have been strengthened by measuring pressure pain threshold of all the investigated anatomical locations and relating this to the manual palpation scores. As manual palpation scores are prone to many errors, the inclusion of a calibrated team of trained examiners may have strengthened the study. Although, the tenderness scale of 'no', 'some' and 'severe' tenderness does not allow for much sensitivity, it is easy to understand and use in practice. However, in hindsight, a greater resolution of the scale may have been valuable. Future validity- and reproducibility-studies should determine whether a higher resolution of the scale is feasible, e.g. a 5-point tenderness scale or a 100 mm VAS scale for perceived tenderness. It should be noted that the low weighted kappa values may result from using manual measurements and four examiners, and not necessarily because of a lack of clustering of tenderness. Referred pain - i.e. pain perceived at a location adjacent or distant from the injury's origin - which is a common phenomenon in relation to neck and head pain [30], may have caused spreading of tenderness. Finally, the inclusion and exclusion criteria of our study confine the external validity to adults with nonspecific neck/shoulder pain without traumatic injuries or other serious chronic disease.

## Conclusion

In conclusion, a high prevalence of tenderness in several anatomical locations of the neck/shoulder complex among adults with nonspecific neck/shoulder pain exists. Our results indicate that future research on neck/shoulder pain should focus on several muscles, including the levator scapulae, neck extensors and infraspinatus, and not only the upper trapezius.

## Competing interests

The authors declare that they have no competing interests.

## Authors' contributions

LLA and MKZ designed the study. KLH and OSM contributed to the clinical examination. LLA performed the statistical analyses and wrote the draft of the paper, and all authors read and approved the final version.

## Funding

Author LLA received a grant from the Danish Rheumatism Association (grant R68-A993) for this study.

## Pre-publication history

The pre-publication history for this paper can be accessed here:

http://www.biomedcentral.com/1471-2474/12/169/prepub

## References

[B1] Janwantanakul P Pensri P Jiamjarasrangsri V Sinsongsook T Prevalence of self-reported musculoskeletal symptoms among office workers Occup Med (Lond) 2008 12 436 438 10.1093/occmed/kqn072 18544589

[B2] Andersen LL Mortensen OS Hansen JV Burr H A prospective cohort study on severe pain as a risk factor for long-term sickness absence in blue- and white-collar workers Occup Environ Med 2010 10.1136/oem.2010.056259 21071754

[B3] Blangsted AK Sogaard K Hansen EA Hannerz H Sjogaard G One-year randomized controlled trial with different physical-activity programs to reduce musculoskeletal symptoms in the neck and shoulders among office workers Scand J Work Environ Health 2008 12 55 65 18427699 10.5271/sjweh.1192

[B4] Buckle P Ergonomics and musculoskeletal disorders: overview Occup Med (Lond) 2005 12 164 167 10.1093/occmed/kqi081 15857895

[B5] Blangsted AK Hansen K Jensen C Muscle activity during computer-based office work in relation to self-reported job demands and gender Eur J Appl Physiol 2003 12 352 358 10.1007/s00421-003-0805-7 12736845

[B6] Larsson B Sogaard K Rosendal L Work related neck-shoulder pain: a review on magnitude, risk factors, biochemical characteristics, clinical picture and preventive interventions Best Pract Res Clin Rheumatol 2007 12 447 463 10.1016/j.berh.2007.02.015 17602993

[B7] Kaergaard A Andersen JH Rasmussen K Mikkelsen S Identification of neck-shoulder disorders in a 1 year follow-up study. Validation Of a questionnaire-based method Pain 2000 12 305 310 10.1016/S0304-3959(00)00261-X 10812260

[B8] Juul-Kristensen B Kadefors R Hansen K Bystrom P Sandsjo L Sjogaard G Clinical signs and physical function in neck and upper extremities among elderly female computer users: the NEW study Eur J Appl Physiol 2006 12 136 145 10.1007/s00421-004-1220-4 16328188

[B9] Andersen LL Kjaer M Sogaard K Hansen L Kryger AI Sjogaard G Effect of two contrasting types of physical exercise on chronic neck muscle pain Arthritis Rheum 2008 12 84 91 10.1002/art.23256 18163419

[B10] Andersen LL Holtermann A Jorgensen MB Sjogaard G Rapid muscle activation and force capacity in conditions of chronic musculoskeletal pain Clin Biomech (Bristol, Avon) 2008 12 1237 1242 10.1016/j.clinbiomech.2008.08.002 18835071

[B11] Andersen LL Nielsen PK Sogaard K Andersen CH Skotte J Sjogaard G Torque-EMG-velocity relationship in female workers with chronic neck muscle pain J Biomech 2008 12 2029 2035 10.1016/j.jbiomech.2008.03.016 18460408

[B12] Andersen LL Suetta C Andersen JL Kjær M Sjøgaard G Increased proportion of megafibers in chronically painful muscles Pain 2008 12 588 593 10.1016/j.pain.2008.06.013 18701218

[B13] Mackey AL Andersen LL Frandsen U Suetta C Sjogaard G Distribution of myogenic progenitor cells and myonuclei is altered in women with vs. those without chronically painful trapezius muscle J Appl Physiol 2010 10.1152/japplphysiol.00789.2010 20930124

[B14] Rosendal L Larsson B Kristiansen J Peolsson M Sogaard K Kjaer M Sorensen J Gerdle B Increase in muscle nociceptive substances and anaerobic metabolism in patients with trapezius myalgia: microdialysis in rest and during exercise Pain 2004 12 324 334 10.1016/j.pain.2004.09.017 15561388

[B15] Ohlsson K Attewell RG Johnsson B Ahlm A Skerfving S An assessment of neck and upper extremity disorders by questionnaire and clinical examination Ergonomics 1994 12 891 897 10.1080/00140139408963698 8206057

[B16] Andersen LL Saervoll CA Mortensen OS Poulsen OM Hannerz H Zebis MK Effectiveness of small daily amounts of progressive resistance training for frequent neck/shoulder pain: randomised controlled trial Pain 2010 10.1016/j.pain.2010.11.016 21177034

[B17] Pincus T Bergman M Sokka T Roth J Swearingen C Yazici Y Visual analog scales in formats other than a 10 centimeter horizontal line to assess pain and other clinical data J Rheumatol 2008 12 1550 1558 18597409

[B18] Andersen LL Kjaer M Sogaard K Hansen L Kryger AI Sjogaard G Effect of two contrasting types of physical exercise on chronic neck muscle pain Arthritis Rheum 2008 12 84 91 10.1002/art.23256 18163419

[B19] Kuorinka I Jonsson B Kilbom Å Vinterberg H Biering-Sørensen F Andersson G Jørgensen K Standardised Nordic questionnaires for the analysis of musculoskeletal symptoms Appl Ergo 1987 12 233 237 10.1016/0003-6870(87)90010-X 15676628

[B20] Kelly SM Brittle N Allen GM The value of physical tests for subacromial impingement syndrome: a study of diagnostic accuracy Clin Rehabil 2010 12 149 158 10.1177/0269215509346103 20103576

[B21] Tong HC Haig AJ Yamakawa K The Spurling test and cervical radiculopathy Spine (Phila Pa 1976) 2002 12 156 159 10.1097/00007632-200201150-00007 11805661

[B22] Andersen LL Saervoll CA Mortensen OS Poulsen OM Hannerz H Zebis MK Effectiveness of small daily amounts of progressive resistance training for frequent neck/shoulder pain: randomised controlled trial Pain 2011 12 440 446 10.1016/j.pain.2010.11.016 21177034

[B23] Bojsen-Møller F Bevægeapparatets anatomi 2003 København: Munksgaard Danmark

[B24] Veeger HE van der Helm FC Shoulder function: the perfect compromise between mobility and stability J Biomech 2007 12 2119 2129 10.1016/j.jbiomech.2006.10.016 17222853

[B25] Birch L Juul-Kristensen B Jensen C Finsen L Christensen H Acute response to precision, time pressure and mental demand during simulated computer work Scand J Work Environ Health 2000 12 299 305 10994795 10.5271/sjweh.546

[B26] Henriksson KG Hypersensitivity in muscle pain syndromes Curr Pain Headache Rep 2003 12 426 432 10.1007/s11916-003-0058-5 14604501

[B27] Chesterton LS Barlas P Foster NE Baxter GD Wright CC Gender differences in pressure pain threshold in healthy humans Pain 2003 12 259 266 10.1016/S0304-3959(02)00330-5 12583868

[B28] Binderup AT Arendt-Nielsen L Madeleine P Pressure pain sensitivity maps of the neck-shoulder and the low back regions in men and women BMC Musculoskelet Disord 2010 12 234 10.1186/1471-2474-11-234 20939890 PMC2964538

[B29] Ge HY Madeleine P Cairns BE Arendt-Nielsen L Hypoalgesia in the referred pain areas after bilateral injections of hypertonic saline into the trapezius muscles of men and women: a potential experimental model of gender-specific differences Clin J Pain 2006 12 37 44 10.1097/01.ajp.0000149799.01123.38 16340592

[B30] Fernandez-de-las-Penas C Simons D Cuadrado ML Pareja J The role of myofascial trigger points in musculoskeletal pain syndromes of the head and neck Curr Pain Headache Rep 2007 12 365 372 10.1007/s11916-007-0219-z 17894927

